# Supplementary material for: Impact of gender on mid-term prognosis of patients undergoing coronary artery bypass grafting
Source: PLoS One. 2023 Mar 2;18(3):e0279030. doi: 10.1371/journal.pone.0279030 (PMC9980750; doi:10.1371/journal.pone.0279030)
Supplement: S1 Table — (DOC) [file pone.0279030.s001.doc]

| **S1 Table. Clinical outcomes at 5 years in patients treated with isolated CABG** | | | | | | | | |
| --- | --- | --- | --- | --- | --- | --- | --- | --- |
|  | **Female** | **Male** | Univariate analysis | | *P*-value | ¹Multivariate analysis | | *P*-value |
|  |  |  | HR | 95% CI |  | HR | 95% CI |  |
|  | n = 1544 | n = 4645 |  | | |  | | |
| Cardiovascular death or myocardial infarction | 62 (4.0) | 155 (3.3) | 1.22 | 0.91 - 1.64 | 0.185 | 0.99 | 0.72 - 1.37 | 0.953 |
| Cardiovascular death | 51 (3.3) | 121 (2.6) | 1.29 | 0.93 - 1.78 | 0.133 | 0.96 | 0.67 - 1.37 | 0.831 |
| All-cause death | 90 (5.8) | 242 (5.2) | 1.14 | 0.89 - 1.45 | 0.302 | 0.86 | 0.67 - 1.12 | 0.272 |
| Myocardial infarction | 15 (1.0) | 42 (0.9) | 1.09 | 0.61 - 1.97 | 0.772 | 1.14 | 0.59 - 2.20 | 0.695 |
| Stroke | 31 (2.0) | 103 (2.2) | 0.91 | 0.61 - 1.36 | 0.657 | 0.84 | 0.55 - 1.31 | 0.448 |
| BARC type 3-5 bleeding | 38 (2.5) | 115 (2.5) | 1.05 | 0.74 - 1.50 | 0.796 | 0.9 | 0.61 - 1.32 | 0.578 |
| ²MACE | 94 (6.1) | 260 (5.6) | 1.05 | 0.86 - 1.29 | 0.610 | 0.87 | 0.70 - 1.09 | 0.874 |
| Values are n (%). Cumulative incidence of events was presented as Kaplan–Meier estimates. | | | | | | | | |
| ACE = angiotensin converting enzyme, ARB = angiotensin receptor blocker, BARC = bleeding academic research consortium, CABG = coronary artery bypass grafting surgery, CI = confidence interval, HR = hazard ratio, LITA = left internal thoracic artery, MACE = major adverse cardiovascular event, MI = myocardial infarction, RITA = right internal thoracic artery, SVG = saphenous vein graft. | | | | | | | | |
| ¹Adjusted variables included age, body mass index, hypertension, diabetes mellitus, current smoking, heart failure, previous history of MI, use of antiplatelet, use of beta-blocker, use of ACE inhibitor or ARB, use of statin, multi-vessel disease, left main involvement, off-pump CABG, combined valvular surgery, number of anastomosis, use of LITA, use of RITA, use of bilateral thoracic arteries, and use of SVG. | | | | | | | | |
| ²MACE was defined as the composite of cardiovascular death, myocardial infarction, and stroke. | | | | | | | | |
