# Supplementary figures and images for: Impact of gender on mid-term prognosis of patients undergoing coronary artery bypass grafting
Source: PLoS One. 2023 Mar 2;18(3):e0279030. doi: 10.1371/journal.pone.0279030 (PMC9980750; doi:10.1371/journal.pone.0279030)

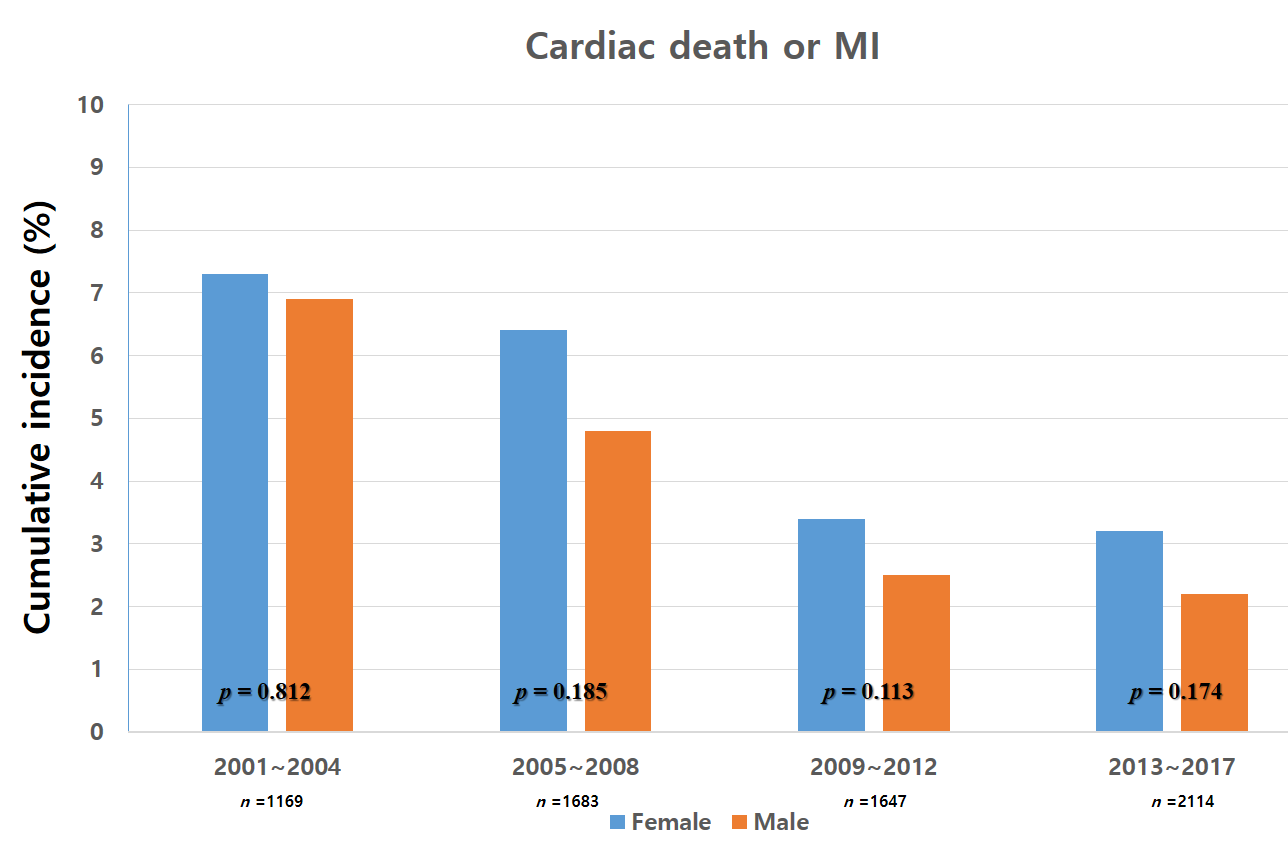

Supplement: S1 Fig — (TIFF) [file pone.0279030.s002.tiff]
