# Supplementary material for: Impact of gender on mid-term prognosis of patients undergoing coronary artery bypass grafting
Source: PLoS One. 2023 Mar 2;18(3):e0279030. doi: 10.1371/journal.pone.0279030 (PMC9980750; doi:10.1371/journal.pone.0279030)
Supplement: Attachment — Submitted filename: Response to Reviewers_PONE-D-22-08451R1.doc [file pone.0279030.s004.doc]

**RESPONSE TO REVIEWERS**

**Reviewer #1's Comment to the Author:**

**Reviewer #1: Have done well to use Propensity Score Matching (PSM) as a tool to study the influence of Sex/Gender on the short and longer term outcome following CABG. A number of PSM studies agree with your finds while others refute it. More recently Chang F-C, et al. BMJ Open 2022;12:e058538 suggests that Female Sex still has a significantly negative impact on the outcome of CABG as well as other major cardiac surgery in a larger cohort of patients (more than 60,000 cases).**

**Also review ... Alam et al. Am J Cardiology. 2013 Aug 1;112(3):309-17**

**…. women who underwent isolated CABG experienced higher mortality at short-term, midterm, and long-term follow-up compared with men. Mortality remained independently associated with female gender despite propensity score-matched analysis of outcomes….**

**And Guadino et al EHJ. 2021 Dec 28;43(1):18-28.**

**→Response to Reviewer #1 Comments**

First of all, I behalf of all authors thank the reviewer #1 for positive comments.

| **Response Table. Comparisons of All-cause mortality after isolated CABG in studies** | | | | | | | | |
| --- | --- | --- | --- | --- | --- | --- | --- | --- |
| **Study** | **Study duration** | **Type of Analysis** | **Clinical outcome** | **Total population** | **Women (n/N, %)** | **Men (n/N, %)** | **adjusted HR (95% CI)** | ***p* value** |
| **Jang et al. (PONE-D-22-08451, The present study)** | 2001 ~ 2017 | PS-matched analysis | 1-year cardiac mortality | 6613 | 66/1679, 6.1% | 134/4934, 4.4% | 1.14 (0.78 - 1.67) | 0.507 |
|  |  |  | 1-year all-cause mortality |  | 116/1679, 10.9% | 272/4934, 8.7% | 1.02 (0.77 - 1.34) | 0.904 |
| Alam et al. Am J Cardiology 2013 Aug 1; 112(3):309-17 | 1982 ~ 2012 | PS- matched meta-analysis | 1-year all-cause mortality | 92660 | 504/19780, 2.6% | 1420/72880, 1.9% | 1.31 (1.18 - 1.45) | 0.710 |
| Guadino et al. EHJ. 2021 Dec 28;43(1):18-28 | 2001 ~ 2012 | Multivariable adjusted  Cox meta-analysis | 5-year all-cause mortality | 13193 | 558/2714, 20.6% | 1817/10479, 17.3% | 1.03 (0.94 - 1.14) | 0.510 |
| Chang F-C et al. BMJ Open 2022;12:e058538 | 2000 ~ 2013 | PS-matched analysis | Long-term all-cause mortality | 42125 | 4380/18720, 44.6% | 4453/18720, 45.4% | 0.98 (0.95 - 1.02) | 0.429 |
| **CABG = coronary artery bypass grafting surgery, CI = confidence interval, HR = hazard ratio, PS = propensity score.** | | | | | | | | |

As the reviewer #1’s comment, the prognostic effect of sex in patients treated with isolated coronary artery bypass grafting (CABG) still is controversy. Chang F-C at al. evaluated the sex-related differences in clinical outcomes after major adult cardiac surgery using nationwide, population-based cohort study [1]. They reported that female patients who underwent CABG (with or without concurrent valvular intervention) generally had worse in-hospital complication (composite of in-hospital mortality, new-onset stroke and new onset dialysis during the admission for the index surgery) and late outcomes (MACE; composite of cardiovascular death, revascularization, acute myocardial infraction or ischaemic stroke during follow-up) but in the isolated CABG patients, they showed no difference in long-term all-cause mortality between women and men same as the results of our study **(Response Table)**. Alam et al. also reported that women who underwent isolated CABG experienced higher mortality at short-term, midterm, and long-term follow-up compared with men but most of their data are very old, including even data before 1990 [2], therefore their conclusion cannot reflect current clinical practice. Especially, because of the incidence of very low incidences of all clinical outcomes **(Response Table)**, the large differences in baseline characteristics between the genders, and suboptimal risk adjustment due to low-quality data, the Alam’s study has considerable limitations. Guadino et al. reported worse MACCE (composite of all-cause mortality, myocardial infarction, stroke, and repeat revascularization) in women over 5 years of follow-up compared to men [3] however they also showed similar all-cause mortality between men and women (adjusted HR 1.03, 95% CI 0.94–1.14; *p* = 0.510) **(Response Table)**. Despite of heterogeneity between several studies about gender disparity, the similarity in long-term mortality is consistent between female and male patients undergoing isolated CABG as in our study. Based on our results and former studies, sex does not seem to influence long-term clinical outcomes, especially long-term mortality in patients who undergo CABG. Further understanding the gender differences in demographics and surgical characteristics can narrow the gap of clinical outcomes and improve their prognosis.

To respond the reviewer #1’s comment, we revised references about prognostic role of gender disparity in CABG and added following new references in the revised manuscript.

(INTRODUCTION, page 4, line 8 and REFERENCES, page 13, line 20-25 in the revised manuscript)

**8. Gaudino M, Di Franco A, Alexander JH, Bakaeen F, Egorova N, Kurlansky P, et al. Sex differences in outcomes after coronary artery bypass grafting: a pooled analysis of individual patient data. Eur Heart J. 2021;43(1):18-28. Epub 2021/08/03. doi: 10.1093/eurheartj/ehab504. PubMed PMID: 34338767; PubMed Central PMCID: PMCPMC8851663.**

**9. Chang FC, Chen SW, Chan YH, Lin CP, Wu VC, Cheng YT, et al. Sex differences in risks of in-hospital and late outcomes after cardiac surgery: a nationwide population-based cohort study. BMJ Open. 2022;12(2):e058538. Epub 2022/02/04. doi: 10.1136/bmjopen-2021-058538. PubMed PMID: 35110325; PubMed Central PMCID: PMCPMC8811586.**

We would like to sincerely thank reviewer #1 for valuable comments. Addressing them fully has significantly strengthened the manuscript.

**REFERENCES**
