# Supplementary material for: Impact of gender on mid-term prognosis of patients undergoing coronary artery bypass grafting
Source: PLoS One. 2023 Mar 2;18(3):e0279030. doi: 10.1371/journal.pone.0279030 (PMC9980750; doi:10.1371/journal.pone.0279030)
Supplement: Attachment — Submitted filename: Response to Reviewers_PONE-D-22-08451R2.doc [file pone.0279030.s005.doc]

**- RESPONSE TO REVIEWERS -**

**Reviewer #1: Thank you for the changes you made.**

**Response to Reviewer #1 Comments**

Dear Professor Reida El Oakley FRCS, MD

I behalf of all co-authors sincerely thank you for your positive comments about our work and also thank you again for the constructive and detailed previous review.

**Reviewer #2: The authors attempt to address the controversial issue of impact of gender on long-term prognosis of patients who underwent coronary artery bypass grafting (CABG). They evaluated their institutional database and concluded that after adjusting for baseline differences, sex does not appear to influence long-term risk of cardiovascular death or myocardial infarction (MI) in patients undergoing CABG. In the opinion of this reviewer the quality of the manuscript will be further enhanced if the authors address the following issues.**

**[1] The title needs to be revised. It will be better to rephrase the title as: Impact of gender on mid-term prognosis of patients undergoing isolated coronary artery bypass grafting.**

**[2] Abstract, Objective: preferably 2 to 3 sentences about the targeted patient population, the current management or treatment controversies and the primary objective(s) of the study.**

**[3] Abstract, Methods: Please mention study design.**

**[4] Please exclude patients with combined procedures and only include a pure cohort of patients who underwent isolated CABG.**

**[5] What risk stratification score (EuroSCORE, STS or Parsonnet) was used for this cohort? Please provide this information.**

**[6] The data was censored at 5 years. Generally 5 years is regarded as mid-term outcome. It is important to replace long-term with mid-term in the title and throughout the manuscript to reflect this fact.**

**[7] Was there any missing data? If so how was it addressed?**

**[8] The study period spans 16 years. It is essential to investigate the effect of era of surgery on outcomes.**

**[9] Please add a few sentences about the unanswered questions and future research.**

**Response to Reviewer #2** (**Dr. Shahzad G. Raja)’s Comments**

**[1] The title needs to be revised. It will be better to rephrase the title as: Impact of gender on mid-term prognosis of patients undergoing isolated coronary artery bypass grafting.**

**→Response #1**

As the Reviewer #2’s comment, we modified our full title and short title in the revised manuscript as follows (Title page, page 1, line 1-3 in the revised manuscript).

**Prognostic role of Sex in Coronary Artery Bypass Grafting**

Short title: Sex and Coronary artery bypass grafting

→ **Impact of Gender on mid-term prognosis of patients undergoing Coronary Artery Bypass Grafting**

Short title: **Gender** and Coronary artery bypass grafting

**[2] Abstract, Objective: preferably 2 to 3 sentences about the targeted patient population, the current management or treatment controversies and the primary objective (s) of the study.**

**→Response #2**

As the Reviewer #2’s comment, we added following sentences in the revised manuscript (ABSTRACT, Objectives, page 4, line 3-4 in the revised manuscript).

**Data on gender differences in current management or clinical outcomes after CABG are controversial, and there have been limited data focusing on them**.

**[3] Abstract, Methods: Please mention study design.**

**→Response #3**

As the Reviewer #2’s comment, we added following sentences about the study design in the revised manuscript (ABSTRACT, Methods, page 4, line 5-7 in the revised manuscript).

**Methods:** Between January 2001 and December 2017, 6613 patients who underwent CABG were enrolled and divided into two groups according to sex (female group, n = 1,679 vs. male group, n = 4,934).

→ **Methods:** **This was a retrospective, single-center, observational study.** Between January 2001 and December 2017, 6613 consecutive patients who underwent CABG were enrolled **from an institutional registry of Samsung Medical Center, Seoul, Korea (Clinicaltrials.gov, NCT03870815)** and divided into two groups according to sex (female group, n = 1679 vs. male group, n = 4934).

**[4] Please exclude patients with combined procedures and only include a pure cohort of patients who underwent isolated CABG.**

**→Response #4**

I agree with Reviewer #2’s concern about clinical effect of combined other procedures in patients treated with CABG. However, as we already mentioned in the original manuscript, our study population included patients with coronary artery disease who underwent CABG including only 424 patients (6.4% of total population) treated with combined valvular surgery (Table 2). As the reviewer’s comment, we additionally evaluated data excluding these 424 patients but clinical outcomes in 6189 isolated CABG patients showed as follows.

| **Supplemental table 1. Clinical outcomes at 5 years in patients treated with isolated CABG** | | | | | | | | |
| --- | --- | --- | --- | --- | --- | --- | --- | --- |
|  | **Female** | **Male** | Univariable analysis | | *P*-value | ¹Multivariable analysis | | *P*-value |
|  |  |  | HR | 95% CI |  | HR | 95% CI |  |
|  | n = 1544 | n = 4645 |  | | |  | | |
| Cardiovascular death or myocardial infarction | 62 (4.0) | 155 (3.3) | 1.22 | 0.91 - 1.64 | 0.185 | 0.99 | 0.72 - 1.37 | 0.953 |
| Cardiovascular death | 51 (3.3) | 121 (2.6) | 1.29 | 0.93 - 1.78 | 0.133 | 0.96 | 0.67 - 1.37 | 0.831 |
| All-cause death | 90 (5.8) | 242 (5.2) | 1.14 | 0.89 - 1.45 | 0.302 | 0.86 | 0.67 - 1.12 | 0.272 |
| Myocardial infarction | 15 (1.0) | 42 (0.9) | 1.09 | 0.61 - 1.97 | 0.772 | 1.14 | 0.59 - 2.20 | 0.695 |
| Stroke | 31 (2.0) | 103 (2.2) | 0.91 | 0.61 - 1.36 | 0.657 | 0.84 | 0.55 - 1.31 | 0.448 |
| BARC type 3-5 bleeding | 38 (2.5) | 115 (2.5) | 1.05 | 0.74 - 1.50 | 0.796 | 0.9 | 0.61 - 1.32 | 0.578 |
| ²MACE | 94 (6.1) | 260 (5.6) | 1.05 | 0.86 - 1.29 | 0.610 | 0.87 | 0.70 - 1.09 | 0.874 |
| Values are n (%). Cumulative incidence of events was presented as Kaplan–Meier estimates. | | | | | | | | |
| ACE = angiotensin converting enzyme, ARB = angiotensin receptor blocker, BARC = bleeding academic research consortium, CABG = coronary artery bypass grafting surgery, CI = confidence interval, HR = hazard ratio, LITA = left internal thoracic artery, MACE = major adverse cardiovascular event, MI = myocardial infarction, RITA = right internal thoracic artery, SVG = saphenous vein graft. | | | | | | | | |
| ¹Adjusted variables included age, body mass index, hypertension, diabetes mellitus, current smoking, heart failure, previous history of MI, use of antiplatelet, use of beta-blocker, use of ACE inhibitor or ARB, use of statin, multi-vessel disease, left main involvement, off-pump CABG, combined valvular surgery, number of anastomosis, use of LITA, use of RITA, use of bilateral thoracic arteries, and use of SVG. | | | | | | | | |
| ²MACE was defined as the composite of cardiovascular death, myocardial infarction, and stroke. | | | | | | | | |

There are similar between the results of original study population and isolated CABG subgroup. Current treatment of CABG performed in accordance with relevant standard guidelines [1] frequently involved combined valvular disease. Therefore, we have thought that data of CABG including combined valvular surgery reflect real-world practice better and it is more meaningful to potential your reader.

To respond the Reviewer #2’s comment, we added above table as supplemental table in the revised manuscript (SUPPLEMENTAL MATERIAL, Supplemental table1, page 2 line 1- page 3, line 7 in the revised manuscript).

**REFERENCE**

1. Neumann FJ, Sousa-Uva M, Ahlsson A, Alfonso F, Banning AP, Benedetto U, et al. 2018 ESC/EACTS Guidelines on myocardial revascularization. Eur Heart J. 2019; 40:87-165. https://doi.org/10.1093/eurheartj/ehy394 PMID: 30165437

**[5] What risk stratification score (EuroSCORE, STS or Parsonnet) was used for this cohort? Please provide this information.**

**→Response #5**

This is the important point of view. Unfortunately, due to the retrospective nature of our registry, we do not have detailed data regarding the surgical risk stratification score such as EuroSCORE, STS or Parsonnet. To the Reviewer #2’s comment, we added following comment in our study limitations (DISCUSSION, Study limitations, page 24, line 9 in the revised manuscript).

Second, because of the retrospective nature of our registry, we could not thoroughly identify alterations in post-operative treatment or medical therapy in all of the study patients during follow-up. Moreover, we also did not have any data on factors that could induce menopause such as genetics, immune system disorders, medical procedures, and premature ovarian failure and any information about socioeconomic variables, reproductive history, or behavioral and psychosocial characteristics. Therefore, due to the lack of such data, we were unable to determine whether these factors could play a role in the prognostic differences that were observed.

→Second, because of the retrospective nature of our registry, we could not thoroughly identify **any surgical risk stratification score** and alterations in surgical procedure, post-operative treatment, or medical therapy in all of the study patients during follow-up. Moreover, we also did not have any data on factors that could induce menopause such as genetics, immune system disorders, medical procedures, and premature ovarian failure and any information about socioeconomic variables, reproductive history, or behavioral and psychosocial characteristics. Therefore, due to the lack of such data, we were unable to determine whether these factors could play a role in the prognostic differences that were observed.

**[6] The data was censored at 5 years. Generally 5 years is regarded as mid-term outcome. It is important to replace long-term with mid-term in the title and throughout the manuscript to reflect this fact.**

**→Response #6**

As the Reviewer #2’s comment, we revised “long-term” to “mid-term” in the title, abstract, and text throughout the whole manuscript as follows.

long-term **→mid-term**

(ABSTRACT, Objectives, page 4, line 2 in the revised manuscript), (INTRODUCTION, page 7, line 12 in the revised manuscript), (DISCUSSION, page 21, line 6, line 7, and line 9 in the revised manuscript), (DISCUSSION, page 23, line 8 in the revised manuscript), (DISCUSSION, study limitation, page 24, line 8 in the revised manuscript), and (CONCLUSION, page 25, line 6 and line 7 in the revised manuscript)

**[7] Was there any missing data? If so how was it addressed?**

**→Response #7**

Thank you for your valuable comment. As you know, since this study was conducted from single-center data, missing data could be minimized through a comprehensive chart review. Therefore, no imputation methods were used to infer missing values of baseline variables.

**[8] The study period spans 16 years. It is essential to investigate the effect of era of surgery on outcomes.**

**→Response #8**

This is also the excellent point of view. As the Reviewer #2’ comment, we additionally investigated the gender difference of primary outcome (cardiac death or MI) by era of CABG in our study population.

The incidences of cardiac death or MI after CABG had improved for both female and male group over time but irrespective of the era of CABG, there are no significant differences in the incidence of cardiac death or MI between female and male groups. To respond the Reviewer #2’s comment, we added above figure as supplemental figure (SUPPLEMENTAL MATERIAL, Supplemental figure 1, page 4 line 1-3 in the revised manuscript) and revised sentences in the study limitations of revised manuscript as follows (DISCUSSION, Study limitations, page 24, line 10 in the revised manuscript).

Second, because of the retrospective nature of our registry, we could not thoroughly identify alterations in post-operative treatment or medical therapy in all of the study patients during follow-up. Moreover, we also did not have any data on factors that could induce menopause such as genetics, immune system disorders, medical procedures, and premature ovarian failure and any information about socioeconomic variables, reproductive history, or behavioral and psychosocial characteristics. Therefore, due to the lack of such data, we were unable to determine whether these factors could play a role in the prognostic differences that were observed.

**→**Second, because of the retrospective nature of our registry, we could not thoroughly identify any data about surgical risk and alterations in **surgical procedure**, post-operative treatment, or medical therapy in all of the study patients during follow-up. Moreover, we also did not have any data on factors that could induce menopause such as genetics, immune system disorders, medical procedures, and premature ovarian failure and any information about socioeconomic variables, reproductive history, or behavioral and psychosocial characteristics. Therefore, due to the lack of such data, we were unable to determine whether these factors could play a role in the prognostic differences that were observed.

**[9] Please add a few sentences about the unanswered questions and future research.**

**→Response #9**

As the Reviewer #2’s comment, we revised following sentence for more clear mention about the unanswered question and future research in the revised manuscript (CONCULUSION, page 25, line 8-9 in the revised manuscript).

Further investigation regarding the potential therapeutic implications of these findings should be considered.

→ Further investigation regarding the potential therapeutic implications of these findings **to narrow sex-related prognosis and disparities** should be considered.

We would like to sincerely thank Dr. Shahzad G. Raja for valuable comments. Addressing them fully has significantly strengthened the manuscript.
